# Benefits and challenges of EMR implementations in low resource settings: a state-of-the-art review

**DOI:** 10.1186/s12911-016-0354-8

**Published:** 2016-09-06

**Authors:** Badeia Jawhari, Dave Ludwick, Louanne Keenan, David Zakus, Robert Hayward

**Affiliations:** 1 Department of Medicine, Faculty of Medicine & Dentistry, University of Alberta, Clinical Sciences Building, 8440-112 St NW 5th floor, 5-112E, T6G 2B7 Edmonton, AB Canada; 2 Innovative Canadians for Change, Edmonton, AB Canada; 3 Sherwood Park Primary Care Network, Sherwood Park, AB Canada; 4 Faculty of Community Services, School of Occupational and Public Health, Ryerson University, Toronto, ON Canada

**Keywords:** Electronic medical record, Electronic health record, Implementation, Urban slum, Limited resource setting, Sub-Saharan Africa

## Abstract

**Background:**

The intent of this review is to discover the types of inquiry and range of objectives and outcomes addressed in studies of the impacts of Electronic Medical Record (EMR) implementations in limited resource settings in sub-Saharan Africa.

**Methods:**

A state-of-the-art review characterized relevant publications from bibliographic databases and grey literature repositories through systematic searching, concept-mapping, relevance and quality filter optimization, methods and outcomes categorization and key article analysis.

**Results:**

From an initial population of 749 domain articles published before February 2015, 32 passed context and methods filters to merit full-text analysis. Relevant literature was classified by type (e.g., secondary, primary), design (e.g., case series, intervention), focus (e.g., processes, outcomes) and context (e.g., location, organization). A conceptual framework of EMR implementation determinants (systems, people, processes, products) was developed to represent current knowledge about the effects of EMRs in resource-constrained settings and to facilitate comparisons with studies in other contexts.

**Discussion:**

This review provides an overall impression of the types and content of health informatics articles about EMR implementations in sub-Saharan Africa. Little is known about the unique effects of EMR efforts in slum settings. The available reports emphasize the complexity and impact of social considerations, outweighing product and system limitations. Summative guides and implementation toolkits were not found but could help EMR implementers.

**Conclusion:**

The future of EMR implementation in sub-Saharan Africa is promising. This review reveals various examples and gaps in understanding how EMR implementations unfold in resource-constrained settings; and opportunities for new inquiry about how to improve deployments in those contexts.

## Background

Health Information Systems (HIS), including Electronic Medical Record (EMR) systems, show promise for facilitating health care improvement. Many sub-Saharan African countries recognize this opportunity and actively deploy e-health technologies, including mobile health devices, electronic medical records, electronic health records, and risk surveillance systems. Despite their characterization as developing countries, some have demonstrated leadership through significant investment in recent-generation health information systems. However, relatively little is known about how the HIS promise can be realized in resource-constrained settings, or about the applicability of evidence arising from well-resourced settings. This review examines published reports about how EMRs have been deployed in sub-Saharan African slums, which intervention attributes associate with deployment success, which categories of benefits and harms are observed, what forms of inquiry have been employed, and where important uncertainty remains.

A preliminary search of mainstream bibliographic databases revealed few reports about experiences with EMRs in resource-limited settings. Most focused on *Human Immunodeficiency Virus* (*HIV*) *and Tuberculosis* (*TB*) patient management [1–3]. Little was reported about the effects of EMRs on primary care practices or on general health outcomes in slum settings. Experimental inquiry is rare and the published reports do not provide the level of detail about methods or findings required for systematic review or meta-analytic synthesis methods. Accordingly, this review adopts a “State-of-the-Art” [4] approach; describing what has been published, how insights were derived from observation, and which issues have been explored by what method. The objective is to provide a foundation for the future application of systematic review methods to an expanding literature about EMR impacts in resource-constrained settings.

## Methods

The review was conducted as a step-wise process. First, a general search strategy was derived from a concept map linking question-appropriate concepts. Associated keywords were discovered from multiple search-review cycles in diverse databases. Second, specific search strategies were optimized for each source database. Third, relevance filters were developed and applied to the search results to determine the prevalence of reports addressing specific settings, interventions and outcomes of interest. Fourth, methods descriptors were devised and used to classify the relevant literature. Finally, papers that were both relevant and methodologically credible were reviewed in detail. The overview results are expressed through description and classification of revealed literature, as well as analysis of the content of the selected literature.

A starter list of search concepts reflected key elements of the review objectives. The list was modified as search cycles were conducted and new concepts emerged in retrieved reports, yielding optimized inclusion and exclusion criteria (Appendix 1). These were matched to standardized Medical Subject Heading (MeSH) terms for use in database-specific search strategies (Appendix 2). Publication dates were not specified because a relative paucity of relevant studies and because the recent appearance of EMRs in the settings of interest made the publication date implicit in other search criteria.

Major North American (MEDLINE) and European (EMBASE) citation databases, and one specialty citation database (GLOBAL HEALTH) were searched in addition to the Cochrane database of reviews and the Cochrane controlled trial registry. A general Internet scan was conducted using the Google search engine. The “Grey literature,” including conference proceedings, theses, websites, and government reports, was explored using Google and Google Scholar. Reference lists of retrieved publications were checked for literature not found through searching. All databases were searched from inception through start of February 2015.

There were challenges minimizing false positives while avoiding false negatives associated with search strategies, possibly because key concepts were represented differently in different databases. For example, MEDLINE, GLOBAL HEALTH and the COCHRANE LIBRARY used the “Africa South of the Sahara” instead of “sub-Saharan Africa” found in EMBASE [1, 5]. Terms for digital health records (e.g., “Electronic Health Record,” “Electronic Medical Record,” “Patient Health Record,” etc.) varied widely. Full-text synonym searching proved important in all indexed databases, having the greatest impact on GOOGLE and GOOGLE SCHOLAR performance.

The results of optimized bibliographic searches were combined to constitute the initial “population” of 695 potentially relevant citations. Internet and grey literature searches discovered 54 additional relevant communications. Of the 749 pooled bibliographic and grey literature citations, 738 referenced papers or articles possibly relevant to EMR use in resource-constrained settings. The abstracts of these were passed through more specific setting (country, practice type, intervention type) and methods (literature type, study type, process or outcome focus) filters to yield 96 papers addressing EMR implementation or adoption challenges in resource-constrained parts of sub-Saharan Africa. The introduction, objectives and methods sections were reviewed to re-apply relevance and methods filters, excluding 54 more reports where EMRs were used in mainly in hospitals rather than community-based clinics.

A total of 32 reports remained for comprehensive full-text review. Seven proved a close fit to the review objectives, six about EMR implementations in Kenya and one about Cameroon experiences [6–12]. None specifically addressed EMR implementation challenges in slum settings. Most were published within the previous 5 years and reports published between 2000–2009 were least informative about EMR challenges, most referencing EMR technology new to low resource settings. There were no reports of long-term sustained initiatives. Greater weight was given to findings reported in the last 5 years. The detailed key paper review did not uncover new search concepts, MeSH terms or full-text synonyms, and bibliography searches did not expose reports not already known from the iterative searching described above.

## Results

### Inquiry types

Relevance-filtered publications were grouped by whether they reported original observations or interpreted the observations of others. The primary literature was further subdivided by the type of inquiry used to generate observations (Table 1).

**Table 1:** Relevance-filtered publications grouped by inquiry type

Inquiry type	Citations
Primary	28
Case Reports	21
Implementation focus	16
Adoption focus	5
Program Descriptions	22
EMR deployment benefits, challenges and system design	22
Observational Inquiry	16
- Qualitative Inquiry	0
User perceptions	2
Patient perceptions	1
- Program evaluation	15
Data quality review	3
Appointment management	6
Workflow assessment	2
Time motion study	2
Experimental Inquiry	1
Uncontrolled trials	1
Secondary Literature	17
- Commentary and editorials	6
- Position statements and guidelines	2
- Narrative reviews	7
- Systematic reviews	2

There were 21 case series reports, where a common intervention crossed multiple EMR implementations. Foci of reportage included paper-to-digital record transformation challenges [13], clinician distraction by user interfaces [14], training effects, and determinants of user acceptance [14]. One case series explored hardware and software barriers to implementation, including corrupted files and server failures [14].

Many case studies shared experience-based recommendations about best practices, with a common theme that user involvement increases buy-in before, during and after implementation. Such buy-in is enhanced by EMR customization, sustainable funding and access to a digitally-capable workforce [14]. Implementation opportunities include, for example, loss of paper storage space and improvements in record filing, stock control [15] and report acceptance by government or funding agencies [11, 12, 15]. One case series shared experiences with different open-source EMRs, including financial implications for groups contemplating adoption in resource-limited settings [16].

Of the retrieved observational studies, none used rigorous qualitative research methods. Although survey studies were common [17–19], few described a-priori objectives, how survey question concepts were developed, how instrument validity was established, or how results were interpreted in light of an analytic framework. Thompson et al. (2010) conducted an observational study using data gathered in ethnographic field notes, but did not report an explicit approach to data-abstraction, coding or purposeful analysis of the recorded observations [20]. Rarely were observations captured pre-implementation or in non-implementation settings. Where observational studies claimed overall user satisfaction with EMRs, the authors often did not reconcile this with their own report of user complaints respecting training burdens, loss of productivity and difficulty finding key information [19].

Program evaluations tended to focus on the quality and application of data accrued by EMRs while commenting on operational considerations like error rates, visit duration, appointment no-shows, wait times, clinic efficiency, and fulfillment of service delivery expectations [18, 19, 21, 22]. One evaluation included a formal time-motion study and noted a patient visit duration reduction of about 10 min [18]. Apparently, productivity improvements were associated with less staff time socializing with colleagues [18]. Another program evaluation reported a 30 % reduction in missed appointments, 24 % reduction in erroneous appointments, and an overall reduction in wait times for nurse and lab technician access [21]. An extraordinarily positive program evaluation claimed a reduction of scheduling error rates from 66.5 to 2.1 % [17].

Some observational reports provided detailed descriptions of EMR designs and pilot implementations, focusing on things like data models, software architecture and performance specifications [1, 15–17, 23, 24]. A common theme related to the benefits of open-source systems in resource-limited settings, presumably because lower up-front costs, with many focus on feature customization, local adaptation and hidden costs of adoption [12, 14–16].

No formal clinical trials, where an EMR-exposed group is compared to a suitable control group, were found among relevance and methods-filtered studies. However, a number of before-after time-series comparisons appeared. These tended to examine impacts on resources and barriers to sustainability, such as staffing requirements, employee retention, training needs, hardware reliability and infrastructure requirements [7, 9, 10, 13, 15, 20, 25–27]. There was a tendency to report positive impacts, with unintended negative effects possibly not included in the recorded observations. Positive effects included increased access to Internet information resources [26], quicker retrieval of patient records, timely access to clinical data, more legible documentation and improved quality and safety of care [1, 6, 13, 26, 28].

Seventeen secondary literature reports appeared among relevance-filtered literature, commonly addressing general facilitators and impediments to EMR implementation. Reported success factors include stakeholder engagement in pre-implementation design, building trust among stakeholders, encouraging emergence of local leadership, nurturing embedded champions, and avoiding big staffing changes. Additionally, implementers are encouraged to use existing systems and software, collaborate with other organizations (leveraging resources), invest in backup capacity, audit user actions, provide on-site training and track usage [10, 12, 15, 18, 25].

A unique report explored the ethical ramifications of EMR implementations. The authors lamented a lack of ethically grounded EMR policies in developing countries and cautioned enthusiasts to heed the principle of “do no harm” when navigating change for clinics, staff and patients [29].

### Inquiry topics

Considered together, the filtered literature addressed recurring themes about EMR design, implementation and impact. Topics covered by both primary and secondary literature were categorized into matters of health processes and health outcomes (Table 2).

**Table 2:** Categorization of inquiry topics

Inquiry topic	Citations
Matters of Process	22
- Patient identification	5
- Encounter and patient management	8
- Medication management	2
- Laboratory management	2
- Document and information management	7
- Systems integration	1
- Human resource utilization	7
- Clinic efficiency	3
- Continuity of care	1
- Communications and team relations	2
- Data integrity	3
- Reporting and Analytics	3
- Auditing	1
Matters of Outcome	22
- Chronic disease guideline compliance	2
- HIV/AIDS management compliance	17
- Tuberculosis management compliance	1
- Medication reconciliation	2
- Medical errors	0
- Quality of care	1
- Maternal and child health guideline compliance	1
- Clinical decision support compliance	1

The most commonly emphasized process improvements associated with EMR implementations relate to improved efficiency of time-consuming or error-prone tasks. The most common of these is identity management. Resource-constrained settings often have difficulty consistently identifying patients from visit to visit and from clinic to clinic, with negative impacts on continuity of care. In sub-Saharan Africa, particularly in Kenya, standardized national personal identifiers are rare [7, 8, 18]. Perhaps for this reason, the retrieved literature frequently credited EMRs for introducing identity management [1, 6, 7, 11, 18], with consequent improved clinic encounter management and human resource utlization [8, 14, 19, 20, 24, 30, 31], reduced chart filing times, improved continuity of care [28, 31], reduced data integrity issues [13, 23, 32], and improved accuracy of reports [18, 20, 23]. The reported improvements in tracking of health exposures and outcomes, reductions in inappropriate test duplication and overall improvement in care coordination [1, 6, 7, 11, 18], are all contingent on the ability to retrieve and compare multiple episodes of care for a uniquely identified patient [12].

Effective communication is another common challenge in resource-constrained settings. Even improved legibility of communications can make a difference. One study reported improved clarity of orders and lists post EMR implementation, with particular improvement in prescription management [14]. Others noted increased usefulness of health data associated with EMR structured data entry [17, 33, 34].

Clinics operating in resource-constrained settings are often accountable to diverse government programs, donor organizations and disease-specific grant programs. An important reported process improvement relates to EMR report-generating capabilities which can significantly reduce the time taken to comply with agency reporting requirements [8, 9, 18, 20, 23].

Resource-constrained settings also have difficulty attracting, training and retaining experienced staff; process problems compounded by the need to up-skill and up-manage for transitions from paper to digital processes. The retrieved literature made frequent reference to EMR impacts on human resources. Developing EMR-permissive skills, attitudes and knowledge within a clinic setting is a commonly reported challenge. Involving users early on in the process development is cited as one strategy for enhancing buy-in and increasing systems awareness [9, 20]. One study reported that user empowerment can increase self-esteem and positive views about the EMR-enabled health facility, with spin-off benefits for the community [9].

Health outcomes affected by EMR implementation can be difficult to track for slum clinics. They typically have short-term interactions with clients, little opportunity for follow-up, and outcomes that are hard to measure. Accordingly, the retrieved literature rarely discusses true EMR-associated health outcomes, and tends to emphasize surrogate outcomes like immunization and medication dispensing rates that may be associated with improved health outcomes. However, the effects of clinical decision support [30] on surrogate outcomes, including medication tracking [13, 14], were not widely explored. Little in the retrieved literature addressed chronic disease or functional outcomes [6, 9]. Most available reports focused on HIV/AIDS and TB management, a priority of global granting agencies, with tracking of medication dispensing and side effect monitoring presumed to improve health outcomes.

### Conceptual framework

**Fig. 1:**
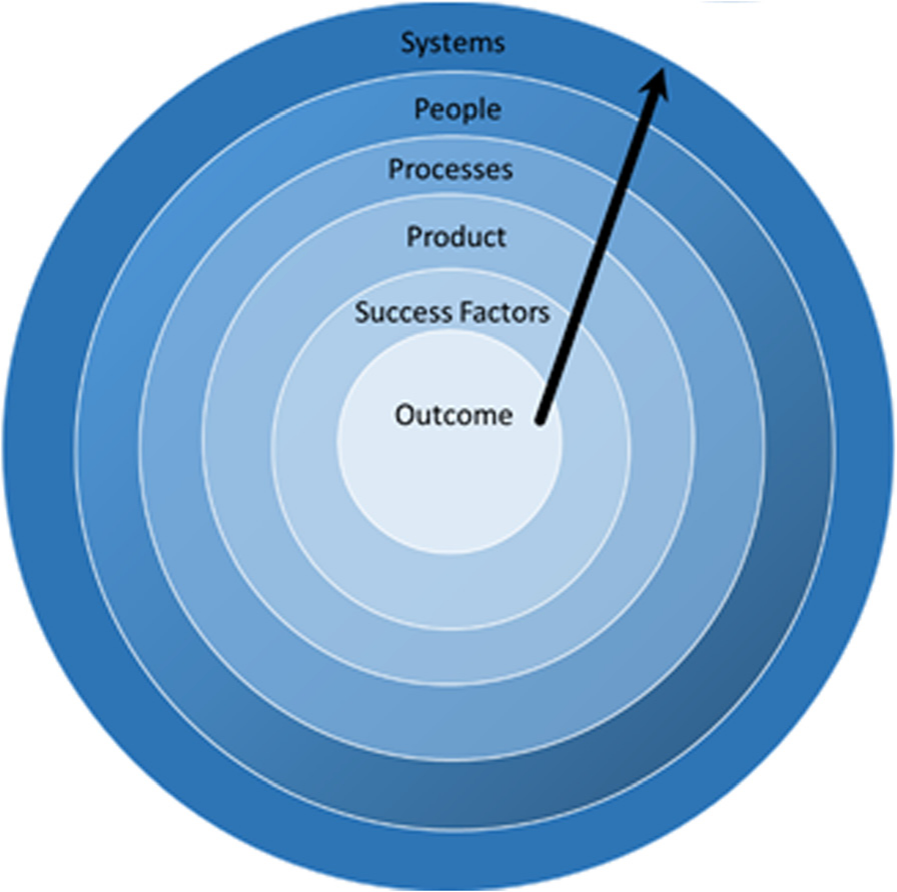
Understanding EMR implementations in limited resource settings

A conceptual framework (Fig. 1) was developed to summarize and synthesize key messages appearing in the discovered literature. This highlights four determinants of EMR impacts in resource-constrained settings: 1) Systems 2) People, 3) Processes, and 4) Products. Systems considerations include access to a reliable power source, suitably located and protected servers and computers, availability of backup systems and the speed and reliability of network and Internet services. People considerations relate to the types of human resources available, how users are trained and supported, how users interact with technology and how users are influenced by workplace attitudes and leadership. Process considerations include change management at the time of deployment and supports post-deployment. Product considerations relate to the electronic medical record software in play and how it inter-operates with other applications. Success factors are those things that authors emphasize as determinants of EMR impact consistent with the goals of EMR implementation.

We suggest that there are no generic facilitators or barriers to EMR implementation applicable in all resource-constrained settings. Rather, the interplay of local systems, people, process and product considerations determine which success factors best predict effective EMR use. This approach is consistent with what the retrieved literature reveals about the variability of facilitators and barriers to effective EMR implementations in resource-constrained settings; and it may suggest an approach to planning future interventions. One might start with a clear statement of desired outcomes, then consider how known success factors must be adapted and prioritized to fit with the local product, process, people and systems opportunities and challenges that will shape implementation experiences (Fig. 1).

### Systems

Many reports highlight the importance of appropriate information systems infrastructure, such as reliable power, connectivity and networking capabilities where EMRs are deployed [7, 8, 11]. Some authors recommend specific remedies for resource-constrained settings, including installation of multiple power supplies of different types (e.g., generator, solar, battery, uninterrupted power supply) to assure continuing hardware and software function [7, 8, 11, 25]. Where mobile EMR products link to centralized information systems, the location of telecommunications towers and service centres become more important considerations.

### People

Socio-technical factors–interactions between patients, providers, staff and their digital environment–are frequently highlighted as powerful determinants of EMR uptake and impact. Typically cited barriers include high staff turnover, absence of local technical support, and low levels of computer literacy. Organizational barriers include lack of local information system leadership or coexistence of multiple co-deployed systems without coordinated leadership [6, 9, 10, 25].

### Processes

Descriptions of process changes, intentional or unintentional, consequential or collateral, figure prominently in the discovered literature. A number of authors observe that EMR implementation does not, by itself, improve the efficiency or effectiveness of health care. Instead, digital systems tend to bring dysfunctional processes into focus, even aggravating bad workflows. One author suggested optimization of paper-based processes as a pre-requisite to EMR implementation [9]. Another suggests that development of structured paper-based data collection forms can help bridge to EMR workflows [7]. Transitional retention of some kind of paper-based workflow can reassure staff that their job will not be replaced or drastically changed post go-live [7].

Some process improvement claims are common. For example, many authors emphasize the importance of user and leadership engagement, noting how training and support can protect against negative reactions to loss of familiar workflows. Achieving meaningful user engagement requires investment pre and post EMR deployment. Although commonly a struggle, user engagement can be facilitated by relatively simple interventions. For example, two reports suggested that providing patients with an identification card (ID) allows them to feel involved in the EMR process, even increasing buy-in because they feel valued [8, 11], possibly by having gained visible badge of association with a prestigeous clinic [11]. Financial incentives can help overcome implementation hurdles for some staff [12]. Details about how the incentives might be matched to performance, and for how long, are scant.

### Products

One report emphasized how limitations of currently available EMR systems contribute to user resistance; especially bugs, missing features and poor performance [10]. Some commonly maligned software features are mandated by government, especially in Kenya. For example, complex security associated with sign-on processes, unrealistically complex reporting requirements, or time-consuming backup rules can be beyond the means of clinics in resource-constrained settings [7, 8]. One study suggests that challenged clinics back up data to paper rather than other electronic systems [7]. Health clinics in resource-constrained settings require well-designed, easy to use EMR software that can be easily customized to the needs of clinic and staff; but the published consensus is that this has yet to be realized.

Improving end-user engagement might, for example, require extraordinary effort to establish rapport with clinic staff, use of multiple site visits to fully understand how workflows and operations must change, strengthen relationships with local partners, find and involve stakeholders, customize product to better fit local needs, and build reliable methods for data collection to track user behavior change [6, 9, 10, 12].

### Outcomes

Desired, expected or achieved outcomes from an EMR implementation vary in the discovered literature. In some resource-limited settings, implementing unique identifiers and improving patient tracking can be sufficient for meeting improved care coordination goals [6, 7]. Other settings seek more complex changes, including the shaping of provider decisions, making clinical decision support an important success factor [6, 30]. Some EMR implementations are credited with tracking patient outcomes, access to a shared medical record, and reduction in medical errors [1, 13]. Others are credited with improved clinic productivity [18] where better appointment management is the outcome dependent on systems, people, processes and product factors. While it is commonly assumed that EMRs should improve health care for patients and populations, some authors contend that this is more likely when research, quality improvement and disease surveillance are explicit goals [11–13].

## Discussion

Overall, available studies of EMR implementation in resource-constrained settings appeared methodologically limited and at an early stage of development. The most relevant reports appeared within the last 5 years, there were no controlled comparison studies, and most communications were descriptive in nature. Hypotheses about factors affecting EMR implementations in slums can be inferred, but they have not been tested. Clear evidence-based recommendations are rare and the prevailing advice is often conflicting. This is understandable given wide variations in the systems, people, process and product factors at play in the settings of interest. To the extent that fundamental infrastructure (e.g., move to wireless and mobile devices), cost (e.g., available open-source EMR software), and support (e.g., emerging information literate workforce) challenges are dynamic, growth in the quantity and quality of relevant literature is anticipated.

Since completing the literature review, two new contributions have appeared [35, 36]. Fritz et al. (2015) examined facilitators, but not barriers, to EMR implementation in limited resource settings [35]. Organizational (e.g., human resource), technical (e.g., infrastructure, Internet and power), functional (e.g., data quality and reporting) and training factors were emphasized, consistent with findings reported here. In addition, “political” and “ethical” factors were observed, which did not figure prominently in the literature considered by this review. A review by Tierney et al. (2015) referenced studies considered in our review, with similar findings [7, 11, 18].

Given the methodological state of the available literature, and the types of questions raised by that literature, it would appear that credible qualitative studies are needed. The human factors affecting EMR uptake in resource-constrained environments are complex. These need to be better characterized before good implementation impact measures are designed, assessment methods are developed, evaluation frameworks validated and comparative studies become doable.

### Limitations

We are aware of a number of limitations to the State-of-the-Art review reported here. The literature retrieval and iterative review process was systematic, but not validated by checks for intra and inter-rater reliability. Portuguese, French and Swahili are commonly spoken in sub-Saharan Africa, but less commonly used for health informatics communications. Nonetheless, it is possible that our English-language constraint missed potentially important articles. Although potentially important research databases, such as CINAHL and INEEX Explorer, were not searched, careful checks of the bibliographies of retrieved papers suggest that the English language literature retrieval was comprehensive for the time period covered.

Limiting the literature search to sub-Saharan African countries may have limited opportunities to capture papers that may have discussed EMR implementations in slum areas elsewhere. Given the breadth of the initial search strategy, and the size of the initial population of studies scanned, the impression is that any missed literature about EMRs in slum settings elsewhere would not be more methodologically mature or likely to yield significantly different insights.

## Conclusion

Systems, people, process and product factors play an integral role in the fate of EMR implementations in sub-Saharan African countries. Increased investment and deployment is likely given growth in multi-institutional collaborations, government support and funding priorities. This State-of-the-Art review identifies both knowledges gaps and learning opportunities for EMR use in resource-limited settings. More consistent and informative reporting about implementation studies could improve our ability to discover the most important determinants of success, and the most important harms to avoid. There is a need for rigorous qualitative research before valid quantitative studies can be contemplated. Comparisons between emerging experiential and experimental reports could be facilitated by reference to a conceptual framework that organizes systems, people, process and product considerations.

## Appendix 1: Literature review properties

### Search checklist

- Include papers that focus on implementation of EMRs and EHRs (some bibliographic databases and countries refer to these two terms interchangeably)
- Include papers that focus on implementation challenges/barriers/disadvantages/failures of EMRs/EHRs/HIS
- Include papers that focus on implementation benefits/advantages/successes of EMRs
- Include papers that focus on implementation of EMRs in low resource settings
- Include papers that focus on implementation of EMRs in slum settings
- Include papers that focus on sub-Saharan Africa and Kenya

### Inclusion criteria

- Include papers that focused on EMRs or EHRs
- Include papers that referred to eHealth systems or health information systems (that focused on EMRs or EHRs)
- Include papers that include low resource settings or limited resource settings
- Include papers that focused on sub-Saharan Africa, African countries
- Include papers that focused on Kenya and developing countries (that focus on sub-Saharan African countries)
- Include papers that focused on implementation of EMRs/EHRs or HIS systems that also referred to challenges/barriers/disadvantages and benefits/advantages of implementation
- Include papers which document the implementation or deployment of EMRs/EHRs/HIS system where authors discuss lessons learned, risks, outcomes or recommendations
- Include papers that include both positive, and negative views/results of implementations
- Include papers that discussed human resources, workflow, patient outcomes, buy-in of stakeholders, staff input or involvement in the implementation process, information technology infrastructure, privacy, confidentiality, safety, user perceptions, user satisfaction/dissatisfaction, successes, failures, adoption, or any other factors that are included in the pre, during, and post, implementation of such systems
- Include both quantitative and qualitative studies
- Include papers that refer to or focus on implementation in slum settings
- Include systematic reviews or literature reviews that align with the inclusion criteria
- Include all papers that meet inclusion criteria regardless of date

### Exclusion criteria

- Exclude papers that did not include resource-limited environments
- Exclude papers that refer to databases or systems that manage health data such as District Health Information System, National Health Information Systems, National EMR/EHR/HIS implementations
- Exclude papers that refer to EHRs/EMRs/HIS implemented in hospital based settings
- Exclude papers that refer to hospital information systems, hospital management information systems, health management information systems, or health information systems implemented in a hospital setting
- Exclude papers that included or solely focused on financial analysis, cost, return on investment of EMRs
- Exclude papers that just focused on computerized physician order entry (CPOE) systems
- Exclude papers that focused on clinical decision support systems or clinical summaries systems
- Exclude papers that exclusively focused on electronic personal health records, electronic patient medical records, electronic patient registries, or patient centered health records
- Exclude papers that focused solely on personal digital assistant (PDA)
- Exclude papers that focused on mobile health technologies/systems, handheld technologies/systems/computers and/or mhealth strategies/policies
- Exclude papers on telemedicine
- Exclude papers that only focused on pharmacy stock systems or pharmacy medical systems
- Exclude papers on ambulance systems or ambulatory care systems
- Exclude papers on diabetes management systems or chronic disease management systems
- Exclude paper on laboratory information systems or laboratory management information systems
- Exclude papers on mental health systems or mental health information/tracking systems
- Exclude papers on medication management systems or medication therapy management systems
- Exclude paper that focus on immunization based systems
- Exclude papers that focus on dental information systems or dental health systems
- Exclude papers that focus on obstetrics health information systems
- Exclude papers that focus on occupational health information systems
- Exclude papers that focus on animal management information systems, animal health information systems and animal tracking/surveillance systems
- Exclude papers on data management systems, nutrition information systems, reporting systems, surveillance systems or emergency based data health systems that did not refer to EMRs or EHRs
- Exclude papers that focus on EMR/EHR/HIS implementations in developed countries or countries not in sub-Saharan Africa

## Appendix 2: Search strategy

### Medline

Ovid MEDLINE (R) In-Process & Other Non-Indexed Citations and Ovid MEDLINE (R) 1946 to Present.

Search Terms:

1. computerized medical record system.mp. or exp Medical Records Systems, Computerized/
2. (electronic medical record* or electronic health record* or emr or ehr).mp. [mp = title, abstract, original title, name of substance word, subject heading word, keyword heading word, protocol supplementary concept word, rare disease supplementary concept word, unique identifier]
3. exp Health Information Systems/
4. 1 or 2 or 3
5. Developing Countries/
6. exp Poverty Areas/ or exp Poverty/
7. exp “Africa South of the Sahara”/
8. (low resource* or limited resource* or low income or resource poor or poverty or developing countr* or developing world).mp. [mp = title, abstract, original title, name of substance word, subject heading word, keyword heading word, protocol supplementary concept word, rare disease supplementary concept word, unique identifier]
9. 5 or 6 or 7 or 8
10. 4 and 9
11. (adopt* or implement* or uptake or challeng* or benefit* or barrier*).mp. [mp = title, abstract, original title, name of substance word, subject heading word, keyword heading word, protocol supplementary concept word, rare disease supplementary concept word, unique identifier]
12. 10 and 11
13. (Nairobi or Kenya).mp. [mp = title, abstract, original title, name of substance word, subject heading word, keyword heading word, protocol supplementary concept word, rare disease supplementary concept word, unique identifier]
14. 4 and 13
15. 12 or 14

### Embase

(1974 to 2014 December 19)

Search Terms:

1. electronic medical record/
2. electronic medical record*.mp. [mp = title, abstract, subject headings, heading word, drug trade name, original title, device manufacturer, drug manufacturer, device trade name, keyword]
3. electronic health record*.mp. [mp = title, abstract, subject headings, heading word, drug trade name, original title, device manufacturer, drug manufacturer, device trade name, keyword]
4. exp medical information system/
5. 1 or 2 or 3 or 4
6. low resource*.mp. [mp = title, abstract, subject headings, heading word, drug trade name, original title, device manufacturer, drug manufacturer, device trade name, keyword]
7. resource poor.mp. [mp = title, abstract, subject headings, heading word, drug trade name, original title, device manufacturer, drug manufacturer, device trade name, keyword]
8. limited resources.mp. [mp = title, abstract, subject headings, heading word, drug trade name, original title, device manufacturer, drug manufacturer, device trade name, keyword]
9. marginalized population*.mp. [mp = title, abstract, subject headings, heading word, drug trade name, original title, device manufacturer, drug manufacturer, device trade name, keyword]
10. low income.mp. [mp = title, abstract, subject headings, heading word, drug trade name, original title, device manufacturer, drug manufacturer, device trade name, keyword]
11. exp developing country/
12. poverty/
13. (developing countr* or developing world).mp. [mp = title, abstract, subject headings, heading word, drug trade name, original title, device manufacturer, drug manufacturer, device trade name, keyword]
14. exp “Africa south of the Sahara”/
15. 6 or 7 or 8 or 9 or 10 or 11 or 12 or 13 or 14
16. (adopt* or implement* or uptake).mp. [mp = title, abstract, subject headings, heading word, drug trade name, original title, device manufacturer, drug manufacturer, device trade name, keyword]
17. (challeng* or barrier* or benefit*).mp. [mp = title, abstract, subject headings, heading word, drug trade name, original title, device manufacturer, drug manufacturer, device trade name, keyword]
18. 5 and 15 and (16 or 17)
19. Nairobi.mp. [mp = title, abstract, subject headings, heading word, drug trade name, original title, device manufacturer, drug manufacturer, device trade name, keyword]
20. Kenya.mp. [mp = title, abstract, subject headings, heading word, drug trade name, original title, device manufacturer, drug manufacturer, device trade name, keyword]
21. 19 or 20
22. 5 and 21
23. 18 or 22

### Global health

(1910–2014 Week 50)

Search Terms:

1. Medical Records Systems.mp.
2. electronic medical record*.mp. [mp = abstract, title, original title, broad terms, heading words, identifiers, cabicodes]
3. electronic health record*.mp. [mp = abstract, title, original title, broad terms, heading words, identifiers, cabicodes]
4. health information system*.mp. [mp = abstract, title, original title, broad terms, heading words, identifiers, cabicodes]
5. 1 or 2 or 3 or 4
6. low resource*.mp. [mp = abstract, title, original title, broad terms, heading words, identifiers, cabicodes]
7. resource poor.mp. [mp = abstract, title, original title, broad terms, heading words, identifiers, cabicodes]
8. limited resources.mp. [mp = abstract, title, original title, broad terms, heading words, identifiers, cabicodes]
9. marginalized population*.mp. [mp = abstract, title, original title, broad terms, heading words, identifiers, cabicodes]
10. low income.mp. [mp = abstract, title, original title, broad terms, heading words, identifiers, cabicodes]
11. Developing Countries/
12. exp Poverty/
13. exp “Africa South of Sahara”/
14. (developing countr* or developing world).mp. [mp = abstract, title, original title, broad terms, heading words, identifiers, cabicodes]
15. 6 or 7 or 8 or 9 or 10 or 11 or 12 or 13 or 14
16. (adopt* or implement* or uptake).mp. [mp = abstract, title, original title, broad terms, heading words, identifiers, cabicodes]
17. (challeng* or barrier* or benefit*).mp. [mp = abstract, title, original title, broad terms, heading words, identifiers, cabicodes]
18. 16 or 17
19. nairobi.mp. [mp = abstract, title, original title, broad terms, heading words, identifiers, cabicodes]
20. kenya.mp. [mp = abstract, title, original title, broad terms, heading words, identifiers, cabicodes]
21. 5 and 15 and 18
22. 19 or 20
23. 5 and 22
24. 21 or 23

### Cochrane

(All EMB Reviews)

EBM Reviews - Cochrane Database of Systematic Reviews 2005 to November 2014, EBM Reviews-ACP Journal Club 1991 to December 2014, EBM Reviews-Database of Abstracts of Reviews of Effects 4th Quarter 2014, EBM Reviews-Cochrane Central Register of Controlled Trials November 2014, EBM Reviews-Cochrane Methodology Register 3rd Quarter 2012, EBM Reviews-Health Technology Assessment 4th Quarter 2014, EBM Reviews-NHS Economic Evaluation Database 4th Quarter 2014

Search Terms

1. computerized medical record system.mp. or exp Medical Records Systems, Computerized/
2. (electronic medical record* or electronic health record* or emr or ehr).mp. [mp = title, abstract, original title, name of substance word, subject heading word, keyword heading word, protocol supplementary concept word, rare disease supplementary concept word, unique identifier]
3. exp Health Information Systems/
4. 1 or 2 or 3
5. Developing Countries/
6. exp Poverty Areas/ or exp Poverty/
7. exp “Africa South of the Sahara”/
8. (low resource* or limited resource* or low income or resource poor or poverty or developing countr* or developing world).mp. [mp = title, abstract, original title, name of substance word, subject heading word, keyword heading word, protocol supplementary concept word, rare disease supplementary concept word, unique identifier]
9. 5 or 6 or 7 or 8
10. 4 and 9
11. (adopt* or implement* or uptake or challeng* or benefit* or barrier*).mp. [mp = title, abstract, original title, name of substance word, subject heading word, keyword heading word, protocol supplementary concept word, rare disease supplementary concept word, unique identifier]
12. 10 and 11 (total papers 206)
13. (Nairobi or Kenya).mp. [mp = title, abstract, original title, name of substance word, subject heading word, keyword heading word, protocol supplementary concept word, rare disease supplementary concept word, unique identifier]
14. 4 and 13
15. 12 or 14

### Google Search

Search Strings

1. “electronic medical record system” Kenya
2. (“electronic medical record system” or “electronic health record system” or “health information system”) + (“Kenya” or “Nairobi” or “Africa South”)
3. (“electronic medical record system”) + (“challenge” or “benefit” or “advantages” or “disadvantages” or “implement” or “deploy” or “adopt”) + (“Nairobi” or “Kenya”)
4. (“electronic medical record system” or “electronic health record system” or “health information system”) + (“challenges” or “benefits” or “advantages” or “disadvantages” or “implement” or “deploy” or “adopt”) + (“limited resource settings” or “limited settings” or “slums” or “poverty areas”)
5. (“electronic medical record system” or “electronic health record system” or “health information system”) + (“challenges” or “benefits” or “advantages” or “disadvantages” or “implement” or “deploy” or “adopt”) + (“Kenya” or “Nairobi” or “Africa South”) + (“limited resource settings” or “limited settings” or “slums” or “poverty areas”)

### Google scholar Search

Search Strings:

1. (“electronic medical record system” or “electronic health record system” or “health information system”) + (“Kenya” or “Africa South”)
2. (“electronic medical record system” or “electronic health record system” or “health information system”) + (“Kenya”)
3. (“electronic medical record system” or “electronic health record system” or “health information system”) + (“developing countries”)
4. (“electronic medical record system” or “electronic health record system” or “health information system”) + (“developing countries”) + (“challenges” or “benefits” or “advantages” or “disadvantages” or “implement” or “deploy” or “adopt”)
5. (“electronic medical record system” or “electronic health record system” or “health information technology”) + (“developing countries”)
6. (“electronic medical record system” or “health information technology”) + (“developing countries”)
7. (“electronic medical record system”) + (“developing countries”) + (“challenges” or “benefits” or “implement” or “deploy” or “adopt”)
8. (“electronic medical record system”) + (“developing countries”) + (“World Health Organization”)
9. (“electronic medical record system”) + (“developing countries”) + (“United Nations”)

## Abbreviations

EMR: Electronic medical record
HIS: Health information system
HIV: Human immunodeficiency virus
ID: Identification card
MeSH: Medical subject heading
TB: Tuberculosis
